# MAPK1/ERK2 as novel target genes for pain in head and neck cancer patients

**DOI:** 10.1186/s12863-016-0348-7

**Published:** 2016-02-13

**Authors:** Cielito C. Reyes-Gibby, Jian Wang, Mary Rose T. Silvas, Robert Yu, Sai-Ching J. Yeung, Sanjay Shete

**Affiliations:** Department of Emergency Medicine, The University of Texas MD Anderson Cancer Center, Houston, TX 77030 U.S.A.; Department of Biostatistics, The University of Texas MD Anderson Cancer Center, Houston, TX 77030 U.S.A.; Department of Epidemiology, The University of Texas MD Anderson Cancer Center, Houston, TX 77030 U.S.A

**Keywords:** Cancer pain, Head and neck cancer, MAPK1/ERK2, Ingenuity pathway analysis, Gene, SNP

## Abstract

**Background:**

Genetic susceptibility plays an important role in the risk of developing pain in individuals with cancer. As a complex trait, multiple genes underlie this susceptibility. We used gene network analyses to identify novel target genes associated with pain in patients newly diagnosed with squamous cell carcinoma of the head and neck (HNSCC).

**Results:**

We first identified 36 cancer pain-related genes (i.e., focus genes) from 36 publications based on a literature search. The Ingenuity Pathway Analysis (IPA) analysis identified additional genes that are functionally related to the 36 focus genes through pathway relationships yielding a total of 82 genes. Subsequently, 800 SNPs within the 82 IPA-selected genes on the Illumina HumanOmniExpress-12v1 platform were selected from a large-scale genotyping effort. Association analyses between the 800 candidate SNPs (covering 82 genes) and pain in a patient cohort of 1368 patients with HNSCC (206 patients with severe pain vs. 1162 with non-severe pain) showed the highest significance for *MAPK1/ERK2*, a gene belonging to the MAP kinase family (rs8136867, *p* value = 8.92 × 10^−4^; odds ratio [OR] = 1.33, 95 % confidence interval [CI]: 1.13–1.58). Other top genes were *PIK3C2G* (a member of PI3K [complex], rs10770367, *p* value = 1.10 × 10^−3^; OR = 1.46, 95 % CI: 1.16–1.82), *TCRA* (the alpha chain of T-cell receptor, rs6572493, *p* value = 2.84 × 10^−3^; OR = 0.70, 95 % CI: 0.55–0.88), *PDGFC* (platelet-derived growth factor C, rs6845322, *p* value = 4.88 × 10^−3^; OR = 1.32, 95 % CI: 1.09–1.60), and *CD247* (a member of CD3, rs2995082, *p* value = 7.79 × 10^−3^; OR = 0.76, 95 % CI: 0.62–0.93).

**Conclusions:**

Our findings provide novel candidate genes and biological pathways underlying pain in cancer patients. Further study of the variations of these candidate genes could inform clinical decision making when treating cancer pain.

**Electronic supplementary material:**

The online version of this article (doi:10.1186/s12863-016-0348-7) contains supplementary material, which is available to authorized users.

## Background

Head and neck cancer is the sixth most common malignancy worldwide. Squamous cell cancer of the head and neck (HNSCC) is the most common head and neck cancer that includes cancers of the oral cavity (including the gums and tongue), pharynx, and larynx. Relative to other cancers, patients with head and neck cancer have a better prognosis, with overall mortality rates for head and neck cancers declining since 2001 [1]. However, as many as two thirds present with advanced stage of disease and with debilitating symptoms that impacts their quality of life [2]. Therefore, clinical management of symptoms associated with head and neck cancer and cancer treatment is an important goal in managing patients with head and neck cancer.

Pain, which is often the first symptom of head and neck cancer, is prevalent and may be persistent, adversely affecting the quality of life of survivors [2]. Recently, we showed that pain impacts survival [2] in head and neck cancer patients. Therefore, understanding risk factors for pain has huge clinical implications. Our studies and those of others have shown that genetic factors play a key role in vulnerability to pain in cancer patients, and have identified important candidate genes such as opioid receptor genes (e.g., *OPRK1* and *OPRM1*) [3–6], catechol-O-methyltransferase (*COMT*) [7, 8], and cytokine genes [9–17]. These studies mainly focused on specific biological pathways. However, as a complex human trait, it is understood that several genes underlie pain [9, 10, 16, 18, 19] and a comprehensive assessment of genetic risk factors and putative biological pathways for pain is compelling.

The purpose of this study is to identify cancer pain-related genes using a literature search following with the Ingenuity Pathway Analysis (IPA; Ingenuity® Systems, www.ingenuity.com) and then to assess association between the common genetic variants within these IPA-derived genes and cancer related pain in HNSCC patients. Recently, novel network-based approaches have been employed to systematically explore the molecular complexity of diseases [20–24]. Network-based approaches can provide a “big” picture that integrates epidemiological associations with the body of scientific knowledge about complex intracellular and intercellular interactions involved in diseases [23, 25]. Further, network-based approaches have the advantage of identifying disease- or phenotype-related genes and pathways, and in turn, can offer a better understanding of the underlying biological mechanisms [21]. In this study, we used IPA as a bioinformatic tool to synthesize the comprehensive pathway and network analyses of the known genes implicated in cancer pain, which we retrieved from the literature review. The network generated from the IPA core analysis suggests new candidate genes for cancer pain studies. Subsequently, we selected 800 SNPs from a large-scale genotyping effort, genotyped using Illumina HumanOmniExpress-12v1 BeadChip, within the IPA-derived candidate genes and evaluated their association with pre-treatment pain in patients newly diagnosed with squamous cell carcinoma of the head and neck.

## Methods

We first conducted a literature search on genetic studies of pain, as described below. Second, using genes pooled from literature as a starting point, we used IPA to generate gene networks for pain and identified additional genes that are functionally related to the genes obtained from literature search; and finally, we selected SNPs from a large-scale genotyping effort within those IPA-derived genes and assessed the association between the SNPs and pre-treatment pain in 1368 HNSCC patients.

### Literature search

We used the PubMed database to perform a comprehensive literature review, limiting our search to human studies and articles published in English prior to July 2014. The primary purpose of the literature search was to identify genes associated with pain in cancer patients. The genes identified through this search will serve as “focus genes” in the IPA analyses. The search terms used were “cancer pain SNP,” “cancer pain SNPs,” “cancer pain gene,” cancer pain genes,” “cancer chronic pain SNP,” “cancer chronic pain SNPs,” “cancer chronic pain gene,” “cancer chronic pain genes,” “cancer neuropathic pain SNP,” “cancer neuropathic pain SNPs,” “cancer neuropathic pain gene” and “cancer neuropathic pain genes”. Particularly, we used singular and plural keywords separately because we identified additional papers through such search than using only singular keywords. We screened the articles initially identified in our search on the basis of the title, abstract, and full text, and excluded duplicate articles. We then manually searched the reference lists of those articles and of related review articles to identify additional relevant articles (Table 1). From these studies, we retrieved information about genes that harbor or are close to significantly associated genetic variants (SNPs or haplotypes) and included those genes in the IPA. In particular, we included only genes that either have a known biological functional significance (e.g., mediators of the inflammatory response, multi-drug resistance, or drug metabolism) or have been replicated in an independent study.

**Table 1:** Numbers of articles obtained using different search terms

Search terms	# of articles by PubMed search	# of articles by initial screen	# of articles from references	# of articles included
Cancer pain SNPs (SNP)	74	20	14	34
Cancer pain genes (gene)	1207	0	0	0
Cancer neuropathic pain SNPs (SNP)	4	0	0	0
Cancer neuropathic pain genes (gene)	78	2	0	2
Cancer chronic pain SNPs (SNP)	12	0	0	0
Cancer chronic pain genes (gene)	204	0	0	0
Total	1579	22	14	36

### Ingenuity pathway analysis

IPA is a system that connects a list of molecules into a set of networks using the scientific information contained in the Ingenuity Knowledge Base, which is the largest knowledge base of biological interactions and functional annotations [23, 26]. In the networks, nodes are used to represent molecules, which include genes, chemicals, protein families, complexes, microRNA species and biological processes [27]; whereas lines (edges and arrows) connecting two molecules are used to represent relationships between them.

In this study, we utilized the IPA core analysis function to provide interpretation for the genes identified from the literature review (denoted as focus genes in IPA) in the context of biological functions and canonical pathways, as well as to generate relevant networks identifying additional genes that interact with the focus genes. The resulting genes could be considered as candidate genes of interest for future studies of cancer pain. The network generated from IPA analysis also provides a bigger picture about the genes that are likely to be interacting and are directly or indirectly associated with the cancer pain.

The core analysis function in IPA determines biological functions, searches for signaling and metabolic canonical pathways and creates molecule networks on the basis of the focus genes [28]. The biological functions and canonical pathways are based on the literature and are independent of focus genes. The network is created using the focus genes. Each focus gene, irrespective of how many papers reported that gene, is equally weighted in the IPA core analysis. In the IPA core analyses, the key assumption in developing a network is that the biological function involves locally dense interactions [29]. The network generation algorithm involves the following steps: (1) rank the focus genes in a decreasing order based on their connectivity; (2) the most connected focus gene is used as the starting seed gene and a seed gene network is generated using a subset of remaining focus genes that are in the neighborhood of the starting seed gene. A neighborhood is defined as a gene plus the genes exactly one connection away from that gene; (3) generate the second seed gene network using the focus genes not belonging to the first seed gene network. The process continues until all focus genes are included in a seed gene network; (4) connect the seed gene networks through additional non-focus genes; (5) connect additional genes or networks from IPA’s database to the existing network if the network has not reached the maximum pre-specified network size (e.g., 140 genes). Specifically, when identifying additional genes to be added, IPA gives priority to the genes that have the largest overlap with the existing network and have the least number of neighbors. This property is measured using a metric called specific connectivity, which is calculated by dividing the number of genes in the intersection of the neighborhood and the existing network by the union of the number of genes in the neighborhood and the existing network [29]. The gene with the highest specific connectivity score is included in the existing network. Importantly, with the use of this network generation algorithm, the IPA analysis can exclude a focus gene from the resulting network if such a gene is less likely to have connections (i.e., biological relationships) with the network.

The resulting functions/pathways/networks are evaluated using a right-tailed Fisher’s exact test. The *p* values obtained on the basis of this test measure the likelihood that the association between a set of focus genes and a given function/pathway/network is due to random chance [30]. The null hypothesis is that the proportion of the focus genes mapping to a function/pathway/network is similar to the proportion that are mapped in the entire reference set [28]. A score, which is assessed as -log_10_(*p* value), is used to rank the resulting functions/pathways/networks. We used a significance level of <10^−5^ in our study (score > 5) when selecting networks as used in previous studies [23].

In our IPA core analysis, we considered the following settings. We used the Ingenuity Knowledge Base as the reference set. Because our focus was on the genetic studies of cancer pain, we included only genes and not the endogenous chemicals. We used all data sources, including Ingenuity Expert Information and Ingenuity Supported Third Party Information. We limited our analysis to human only studies and included tissues and primary cells. Both direct and indirect relationships were considered for the network analysis. When generating networks, we used the settings of a maximum of 140 genes per network and 25 networks per analysis, because the networks up to 140 genes allow for the possibility that the same network can include all focus genes [24]. Adhering to the hypothesis that highly connected molecules (called hubs) are typically associated with diseases or biological functions in humans [21–24, 29], we reported the most interconnected genes in the networks as the key genes of interest.

### Pain and head and neck cancer genetic association

The study population included adult patients with newly diagnosed, histologically confirmed, previously untreated HNSCC. All patients were self-reported Caucasians. The study was approved by the Institutional Review Board at MD Anderson Cancer Center (MDACC), and all participants provided written informed consent.

Pre-treatment cancer pain was rated using a standardized 11-point numeric scale (0 = “no pain” and 10 = “pain as bad as you can imagine”) [31] at presentation of the patients before initiating cancer therapy. We considered a binary pain phenotype, where cases (severe pain) were individuals with severe pre-treatment pain (score ≥ 7) and controls (non-severe pain) were individuals with non-severe pre-treatment pain (score < 7). The study included 1368 HNSCC patients, with 206 severe pain cases (145 male, 61 female; mean age 57 years, standard deviation [sd] = 12) and 1162 non-severe pain controls (915 males, 247 females; mean age 58 years, sd = 11). Genotyping was conducted at MDACC, using the Illumina HumanOmniExpress-12v1 BeadChip. Samples with SNP call rates <90 % were excluded from the analysis. We included all the SNPs from this chip that were within the newly derived candidate genes in our genetic analyses.

Statistical analyses were conducted using PLINK (v1.07) [32] and R (v2.15) software. A nearest neighbor cluster analysis based on genetic similarity was conducted to identify the clusters of individuals, which was used as a covariate in the association analysis. Deviation from Hardy-Weinberg Proportion (HWP) for each SNP was assessed by a 1 degree-of-freedom *χ*^*2*^ test or Fisher’s exact test, where an expected cell count was < 5. SNPs departing from HWP (*p* value ≤ 10^−6^) and minor allele frequencies (MAF) ≤ 5 % in all samples were excluded from the analysis. The association between each SNP genotype and pre-treatment severe pain status was assessed using multivariable unconditional logistic regression, adjusting for sex and age. We report SNPs with the lowest *p* value belonging to a molecule (a gene or a group of genes) [33–37].

## Results

### Literature review

**Fig. 1:**
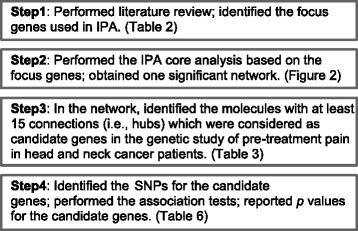
Study flowchart

The overall study flowchart is shown in Fig. 1. Searching the PubMed database using different search terms, we identified 1579 articles. After screening the title, abstract and full text, we excluded 1557 articles for the following reasons (Table 1): (1) not human studies; (2) not published in English; (3) meta-analysis study, review or letter to the editor; (4) clinical trial studies; (5) not genetic association studies; (6) not pain-related phenotypes studies; (7) not cancer patient studies; and (8) duplicate articles from different searches. We then manually searched the reference lists of the 22 articles identified through our search and exclusion criteria and other related review articles about genetic pain studies, and further identified 14 articles. As a result, we had a total of 36 articles from which we identified the genes that will serve as our “focus genes” in order to perform the IPA core analysis (Additional file 1).

The information we retrieved from each of the studies included year of publication, first author, patient ethnicity, cancer type, sample size, phenotypes, and significant genes, which are listed in Additional file 1. These studies included different cancer sites and multiple ethnicities. Different pain-related phenotypes were covered in these studies, such as absolute pain increase between baseline and follow-up, pain intensity before and after opioid administration, contact heat pain and cold pain, persistent postsurgical pain, percentage of pain relief, and aromatase inhibitor-associated musculoskeletal adverse events. We included several studies that employed phenotypes that combined pain measures with other cancer patient symptoms, such as fatigue, depressed mood and morphine side-effect scores, using symptom cluster, a tree-based approach and principal component analysis.

All the 36 articles were association studies between SNPs (or haplotypes) and cancer pain related phenotypes, using either a candidate gene study or a genome-wide association study. From these articles, we identified 36 focus genes eligible for IPA core analysis for pain, which either harbor or are close to the genetic variants (SNPs or haplotypes) found to be statistically significantly associated with the pain-related phenotypes in cancer patients, as listed in Table 2. Some of the genes were implicated in multiple studies. For example, genetic variants within *OPRM1* have been associated with the symptom of cancer pain in four articles [3–6]; and genetic variants within *COMT* have been associated with cancer pain in three articles [7, 8, 38].

**Table 2:** Cancer pain related genes from the literature

Significant genes	References
OPRM1	Klepstad P [3]; Campa D [4]; Droney JM [5]; Ochroch EA [6]
COMT	Hickey OT [7]; Fernández-de-las-Peñas C [8]; Kambur O [38]
IL8	Reyes-Gibby CC [9]; Reyes-Gibby CC [12]; Reyes-Gibby CC [16]
PTGS2	Reyes-Gibby CC [18]; Rausch SM [81]; Reyes-Gibby CC [19]
TNFa	Reyes-Gibby CC [10]; Reyes-Gibby CC [18];
IL10	Rausch SM [13]; Stephens K [17]
CYP19A1	Mao JJ [82]; Garcia-Giralt N [83]
IL4	Illi J [14]; Stephens K [17]
IL1R1	McCann B [15]; Stephens K [17]
IL13	McCann B [15]; Stephens K [17]
ABCB1/MDR1	Campa D [4]
IL6	Reyes-Gibby CC [10]
NFKBIA	Reyes-Gibby CC [18]
TCL1A	Ingle JN [84]
GCH1	Lötsch J [85]
CACNG2	Nissenbaum J [86]
IL1RN	Rausch SM [13]
SPON1	Galvan A [40]
RHBDF2	Galvan A [40]
ZNF235	Galvan A [40]
OPRK1	Droney JM [5]
COX1	Ochroch EA [6]
LTA	Rausch SM [81]
ABCC2	Sloan JA [87]
ABCC4	Sloan JA [87]
CYP17A1	Garcia-Giralt N [83]
VDR	Garcia-Giralt N [83]
CYP27B1	Garcia-Giralt N [83]
ENOS	Reyes-Gibby CC [19]
IL1B	Reyes-Gibby CC [19]
TNFR2	Reyes-Gibby CC [19]
IL10RB	Reyes-Gibby CC [19]
IFNG1	Stephens K [17]
IL1R2	Stephens K [17]
NFKB1	Stephens K [17]
GFRa-2	Wang K [88]

### IPA core analysis

**Fig. 2:**
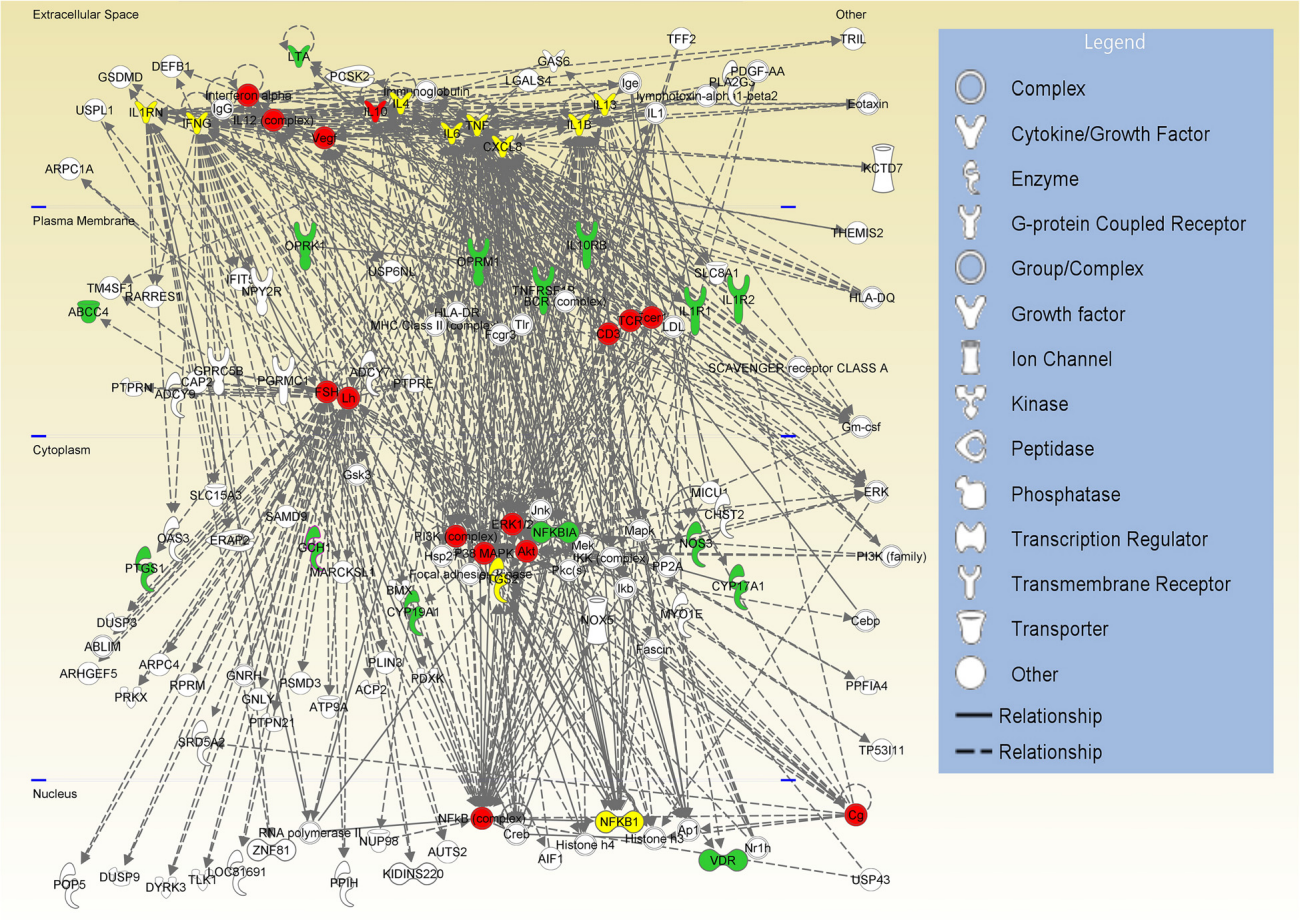
The most significant network (*p* value = 10^−43^) generated by IPA core analysis for cancer pain using 36 focus genes based on cellular locations of the gene products. Green nodes: focus genes with less than 15 connections; red nodes: molecules with at least 15 connections; yellow: focus genes with at least 15 connections. Dashed and solid lines represent indirect and direct interactions, respectively

Six networks were revealed from the IPA core analysis. Based on a nominal significance level of 1 × 10^−5^, only one network was significant (*p* value of 1 × 10^−43^), with 26 of the 36 focus genes (Fig. 2; green: focus genes with less than 15 connections; red: molecules with at least 15 connections; yellow: focus genes with at least 15 connections). In the network, the solid and dashed edges or arrows stand for direct and indirect interactions, respectively. Figure 2 shows the network of focus genes (i.e., known cancer pain-associated genes) and additional non-focus molecules directly or indirectly related to the focus genes.

We are particularly interested in the molecules with most interconnections since it is hypothesized that highly connected molecules are most likely associated with diseases or biological functions [21–24, 29]. Therefore, in Table 3, we reported the top 25 out of 140 molecules that had at least 15 connections (i.e., hubs) in the network shown in Fig. 2 (red and yellow nodes), ranked by the numbers of connections for each of the molecules. The 11 molecules in bold are focus genes. Therefore, we identified 14 additional molecules (most interconnected) that have either direct or indirect interactions with the focus genes obtained from the literature review. These were the candidate molecules of interest in the following genetic association analysis.

**Table 3:** Molecules with at least 15 connections (i.e., hubs) in the network depicted in Fig. 2 (i.e., red and yellow nodes), ranked by the number of connections for each molecule. The molecules in boldface are focus genes

Cancer pain^a^	
IPA symbol	# of connections
**TNF**	**64**
**IFNG**	**49**
**IL1B**	**44**
**CXCL8**	**42**
**IL6**	**39**
Lh	39
FSH	38
NFkB (complex)	38
**IL10**	**31**
P38 MAPK	28
ERK1/2	24
**PTGS2**	**24**
**IL4**	**23**
**IL1RN**	**22**
CD3	21
Vegf	20
**IL13**	**19**
PI3K (complex)	19
IL12 (complex)	17
**NFKB1**	**17**
TCR	17
Akt	16
Fcer1	16
Cg	15
Interferon alpha	15

^a^In the network depicted in Fig. 2, 25 out of 140 molecules have at least 15 connections

In addition to the network, the IPA core analysis also provided the most significant canonical pathways (Table 4) and biological functions (Table 5) across all the focus genes. Table 4 shows the top canonical pathways (*p* value < 1 × 10^−5^) discovered by the IPA core analyses. The significant p-value implies over-representation of focus genes in that pathway. We also calculated ratio of the number of focus genes included in the canonical pathway divided by the total number of genes that make up the canonical pathway. The canonical pathways in Table 4 are ranked by the ratios. The most significant canonical pathway was Hepatic Cholestasis (*p* value = 3.16 × 10^−24^) whereas Airway Inflammation in Asthma had the highest ratio (0.75). In addition, the Table 4 also showed that the identified focus genes are mostly related to cytokine signaling, a pathway that affects the human immune system and inflammation. The proteins expressed by these genes are found in both intra- and extra-cellular matrices. In other words, focus genes that have been connected to cancer pain are not restricted to certain subcellular compartments.

**Table 4:** Top canonical pathways discovered by the IPA core analyses of the focus genes reported to be associated with cancer pain in the literature. The pathways listed have over-representation of focus genes. The canonical pathways were ranked by ratios^a^

Canonical pathways	*p* values	Ratio
Airway Inflammation in Asthma	3.02E-08	75.0 %
Differential Regulation of Cytokine Production in Macrophages and T Helper Cells by IL-17A and IL-17 F	2.09E-10	27.8 %
Differential Regulation of Cytokine Production in Intestinal Epithelial Cells by IL-17A and IL-17 F	8.13E-10	21.7 %
TNFR2 Signaling	2.34E-09	17.9 %
Role of Cytokines in Mediating Communication between Immune Cells	6.31E-16	17.3 %
IL-10 Signaling	7.94E-17	14.7 %
Role of Hypercytokinemia/hyperchemokinemia in the Pathogenesis of Influenza	1.82E-10	14.6 %
T Helper Cell Differentiation	6.31E-13	11.9 %
Graft-versus-Host Disease Signaling	2.51E-08	11.4 %
Altered T Cell and B Cell Signaling in Rheumatoid Arthritis	3.98E-14	11.1 %
IL-6 Signaling	3.16E-18	10.3 %
Hepatic Cholestasis	3.16E-24	10.1 %
PPAR Signaling	1.26E-13	10.0 %
Activation of IRF by Cytosolic Pattern Recognition Receptors	1.95E-09	10.0 %
Role of PKR in Interferon Induction and Antiviral Response	1.20E-06	10.0 %
Communication between Innate and Adaptive Immune Cells	3.98E-12	9.8 %
Role of IL-17 F in Allergic Inflammatory Airway Diseases	1.32E-06	9.8 %
HMGB1 Signaling	3.16E-16	9.3 %
Hematopoiesis from Pluripotent Stem Cells	1.78E-06	9.1 %
TREM1 Signaling	4.68E-09	8.7 %
Role of Pattern Recognition Receptors in Recognition of Bacteria and Viruses	2.51E-14	8.4 %
Allograft Rejection Signaling	2.51E-06	8.3 %
Crosstalk between Dendritic Cells and Natural Killer Cells	4.37E-10	7.9 %
CD40 Signaling	1.74E-07	7.8 %
Type I Diabetes Mellitus Signaling	3.16E-11	7.6 %
IL-15 Signaling	2.00E-07	7.6 %
LXR/RXR Activation	1.58E-12	7.4 %
Role of IL-17A in Arthritis	4.07E-06	7.4 %
Toll-like Receptor Signaling	3.09E-07	6.9 %
Induction of Apoptosis by HIV1	5.75E-06	6.8 %
PXR/RXR Activation	7.59E-06	6.4 %
Hepatic Fibrosis/Hepatic Stellate Cell Activation	2.00E-15	6.1 %
Atherosclerosis Signaling	3.63E-09	5.8 %
Role of Osteoblasts, Osteoclasts and Chondrocytes in Rheumatoid Arthritis	6.31E-15	5.6 %
Dendritic Cell Maturation	3.98E-11	5.3 %
NF-κB Signaling	3.98E-11	5.3 %
p38 MAPK Signaling	1.12E-07	5.1 %
Glucocorticoid Receptor Signaling	1.26E-15	5.0 %
Acute Phase Response Signaling	1.20E-09	4.8 %
LPS/IL-1 Mediated Inhibition of RXR Function	2.29E-10	4.3 %
Role of Macrophages, Fibroblasts and Endothelial Cells in Rheumatoid Arthritis	2.00E-13	4.2 %
Granulocyte Adhesion and Diapedesis	3.31E-08	4.2 %
FXR/RXR Activation	4.90E-06	4.0 %
IL-12 Signaling and Production in Macrophages	6.17E-06	3.8 %
PPARα/RXRα Activation	8.71E-07	3.6 %
Production of Nitric Oxide and Reactive Oxygen Species in Macrophages	1.38E-06	3.4 %

^a^Ratio is calculated as the number of focus genes included in a canonical pathway divided by total number of genes that make up the canonical pathway

**Table 5:** Top 20 diseases and functions discovered by IPA core analyses of focus genes reported to be associated with cancer pain in the literature^a^

Categories	Function annotation	*p* Value
Lipid metabolism, small molecule biochemistry	Synthesis of prostaglandin	1.50E-24
Inflammatory disease	Chronic inflammatory disorder	3.74E-24
Drug metabolism, lipid metabolism, small molecule biochemistry	Synthesis of prostaglandin E2	4.34E-24
Lipid metabolism, molecular transport, small molecule biochemistry	Concentration of eicosanoid	2.19E-21
Gastrointestinal disease, inflammatory disease	Inflammatory bowel disease	1.03E-20
Lipid metabolism, molecular transport, small molecule biochemistry	Concentration of lipid	4.13E-20
Lipid metabolism, small molecule biochemistry	Fatty acid metabolism	6.51E-20
Lipid metabolism, small molecule biochemistry	Synthesis of lipid	1.01E-19
Gastrointestinal disease, inflammatory disease, inflammatory response	Ulcerative colitis	1.74E-19
Connective tissue disorders, immunological disease, inflammatory disease, skeletal and muscular disorders	Rheumatoid arthritis	1.84E-19
Connective tissue, inflammatory disease, skeletal and muscular disorders	Rheumatic disease	4.11E-19
Connective tissue disorders, inflammatory disease, skeletal and muscular disorders	Arthritis	7.15E-19
Inflammatory response	Inflammation of organ	1.19E-18
Immunological disease	Systemic autoimmune syndrome	1.57E-18
Infectious disease	Sepsis	3.20E-18
Inflammatory response	Inflammation of body region	4.40E-17
Lipid metabolism, molecular transport, small molecule biochemistry	Concentration of prostaglandin	4.51E-16
Infectious disease	Dengue hemorrhagic fever	6.48E-16
Inflammatory disease, organismal injury and abnormalities, respiratory disease	Acute respiratory distress syndrome	1.53E-15
Cell-to-cell signaling and interaction, hematological system development and function, immune cell trafficking, inflammatory response	Activation of dendritic cells	2.59E-15

^a^Ranked by *p* values

Table 5 lists the top 20 biological functions discovered by the IPA core analyses, which are ranked using the scores described in Methods section (−log_10_(*p* value)). The top biological functions related to the focus genes are, in general, related to inflammation. The analysis provides a measure of association of focus genes with biological functions. The smaller *p* values imply that the association is non-random. The two most significant biological functions identified through this analysis were lipid metabolism and small molecule biochemistry, and inflammatory disease with *p* values 1.50 × 10^−24^ and 3.74 × 10^−24^, respectively.

### Genetic association between IPA-derived genes and pre-treatment pain in HNSCC patients

We used the 25 most interconnected molecules (i.e., hubs) identified through the IPA core analysis (Fig. 2) as the candidates in the association analysis, as listed in Table 3. Eleven of the 25 candidate molecules were focus genes identified through the literature review. In our study, the candidate molecules identified through IPA core analysis have sub-members (i.e., a group of genes). For example, *Lh*, the luteinizing hormone, has two members, *CGA* and *LHB*, and *CD3* has four members: *CD247*, *CD3D*, *CD3E* and *CD3G*. Also, some genes may belong to more than one molecule. For example, *CGA* belongs to *Lh*, *FSH* and *Cg*. As a result, the 25 most interconnected molecules included a total of 82 genes which were used as the candidate genes in the association analysis. After applying quality control checks, 800 SNPs belonging to the 82 IPA-derived candidate genes were included for the total 1368 HNSCC patients (information for the 82 genes and 800 SNPs is listed in Additional file 2).

The results from the candidate gene association analysis for severe pre-treatment pain in HNSCC patients are shown in Table 6. The first column shows the molecules identified through the IPA analyses, cell location, family, number of SNPs belonging to that molecule in our study, gene name, chromosome location, and the SNP with lowest *p* value belonging to that gene, odds ratio (OR) and *p* value. The gene mitogen-activated protein kinase-1 (*MAPK1*), which belongs to the MAP kinase family and is also known as extracellular signal-regulated protein kinase-2 (*ERK2*), showed the highest significance (rs8136867, *p* value = 8.92 × 10^−4^; OR = 1.33, 95 % confidence interval [CI]: 1.13–1.58). Other genes with *p* values less than 0.01 were *PIK3C2G* (a member of PI3K [complex], rs10770367, *p* value = 1.10 × 10^−3^; OR = 1.46, 95 % CI: 1.16–1.82), *TCRA* (a member of TCR, rs6572493, *p* value = 2.84 × 10^−3^; OR = 0.70, 95 % CI: 0.55–0.88), *PDGFC* (platelet-derived growth factor C, rs6845322, *p* value = 4.88 × 10^−3^; OR = 1.32, 95 % CI: 1.09–1.60), and *CD247* (a member of CD3, rs2995082, *p* value = 7.79 × 10^−3^; OR = 0.76, 95 % CI: 0.62–0.93). These top five genes with germline polymorphisms showing association with pre-treatment pain in the HNSCC patients (Table 6) are listed as non-focus molecules in Table 3. Focus genes in IPA are the genes identified from the literature review as being associated with cancer pain phenotypes. Therefore, the SNPs that we have identified in this study as potentially influencing cancer pain have not been reported elsewhere.

**Table 6:** Results of genetic associated analysis for pre-treatment pain in 1368 head and neck cancer patients (206 severe pain cases and 1162 non-severe pain controls), using the hubs (most interconnected molecules) obtained from IPA core analysis as the candidate molecules. The IPA symbol represents either a gene or a group of genes. The *p* value represents the most significant *p* value within a gene or a gene group. The molecules in boldface are focus genes

IPA symbol	Location	Family	# of SNPs	Genes	Chr	rs#	OR	*P* value
**TNF**	Extracellular space	Cytokine	3	TNF	6	rs1800630	1.20	1.78E-01
**IFNG**	Extracellular space	Cytokine	2	IFNG	12	rs2069727	0.84	8.85E-02
**IL1B**	Extracellular space	Cytokine	4	IL1B	2	rs16944	0.77	3.79E-02
**CXCL8**	Extracellular space	Cytokine	1	CXCL8	4	rs2227543	1.09	3.78E-01
**IL6**	Extracellular space	Cytokine	7	IL6	7	rs2069835	1.45	8.21E-02
Lh	Plasma membrane	Complex	5	CGA	6	rs9359730	1.26	3.48E-02
FSH	Plasma membrane	Complex	4	CGA	6	rs9359730	1.26	3.48E-02
NFkB (complex)	Nucleus	Complex	22	NFKB2	10	rs7897947	1.29	6.78E-02
**IL10**	Extracellular space	Cytokine	4	IL10	1	rs3021094	1.11	6.08E-01
P38 MAPK	Cytoplasm	Group	33	MAPK1	22	rs8136867	1.33	8.92E-04
ERK1/2	Cytoplasm	Group	13	MAPK1	22	rs8136867	1.33	8.92E-04
**PTGS2**	Cytoplasm	Enzyme	3	PTGS2	1	rs5275	1.23	5.35E-02
**IL4**	Extracellular space	Cytokine	5	IL4	5	rs2243248	0.81	3.19E-01
**IL1RN**	Extracellular space	Cytokine	12	IL1RN	2	rs17042917	0.77	1.43E-01
CD3	Plasma membrane	Complex	44	CD247	1	rs2995082	0.76	7.79E-03
Vegf	Extracellular space	Group	47	PDGFC	4	rs6845322	1.32	4.88E-03
**IL13**	Extracellular space	Cytokine	5	IL13	5	rs1881457	0.84	2.44E-01
PI3K (complex)	Cytoplasm	Complex	196	PIK3C2G	12	rs10770367	1.46	1.10E-03
IL12 (complex)	Extracellular space	Complex	10	IL12B	5	rs730691	0.77	1.68E-02
**NFKB1**	Nucleus	Transcription regulator	9	NFKB1	4	rs1609798	1.20	9.42E-02
TCR	Plasma membrane	Complex	378	TCRA	14	rs6572493	0.70	2.84E-03
Akt	Cytoplasm	Group	33	AKT2	19	rs892120	1.35	3.15E-02
Fcer1	Plasma membrane	Complex	15	FCER1G	1	rs11587213	0.68	1.68E-02
Cg	Other	Complex	8	CGA	6	rs9359730	1.26	3.48E-02
Interferon alpha	Extracellular space	Group	14	IFNA7	9	rs4977686	1.20	7.51E-02

## Discussion

Genetic association studies of cancer-related pain have focused on opioid receptors [3–5, 39, 40], *COMT* enzyme [7, 38, 39, 41, 42] and cytokines [10, 12, 16–19, 43]. The primary aim of our study was to identify novel candidate genes for cancer pain through a comprehensive literature search and IPA analysis and then to assess the association between the common genetic variants within these IPA-derived genes and cancer pain in HNSCC patients.

Using genotype data from 1368 HNSCC patients, we found that a germline SNP in *MAPK1* (rs8136867, *p* value = 8.92 × 10^−4^; OR = 1.33, 95 % CI: 1.13–1.58) showed the highest association with cancer pain. *MAPK1* is involved in a number of biochemical signals and cellular processes such as proliferation, differentiation, transcription regulation and development [44]. It was identified as a moonlighting protein [45] because of its ability to act as a transcriptional repressor — an independent and mechanistically distinct function from its kinase activity [46]. It is activated by phosphorylation by an upstream kinase, after which it is translocated to the nucleus to phosphorylate and activate its nuclear substrate [44]. Dysregulation of MAP kinases has been associated with cancer development [47–49]. *MAPK* pathways have also been linked to inflammation [50, 51] and pain [52–56]. The specific *MAPK1* mutation, rs8136867, was reported to be associated with remission in patients with bipolar disorder and major depressive order, possibly having a potential role in neuroplasticity and inflammatory processes [57], and increased risk of developing MSI+ (micro satellite instability) tumor [48]. This increased tumor risk may be directly related to pre-treatment cancer pain since a large tumor can cause pain, especially if it exerts pressure on nearby nerve fibers. None of the genetic association studies for pain in cancer patients (Table 2) used a cohort of patients with HNSCC. Thus, our findings will be the first report on genetic variations that may be relevant to cancer pain in HHSCC patients.

In animal models, various types of nerve injuries in the dorsal root ganglia (DRG) and dorsal horn of the spinal cord have been shown to result in neuropathic pain along with phosphorylation of *MAPK* family such as *ERK* [58–61], *p38* [61–63] and *JNK* [59, 60]. Unlike *p38* and *JNK*, phosphorylation of *ERK* due to nerve injury occurs early and lasts long [64]. *MEK* is an upstream kinase in the *ERK*/*MAPK* pathway. In animal models of neuropathic pain, *MEK* inhibitors have been shown to be effective in alleviating pain at numerous time points [64], suggesting that the regulation of *ERK*/*MAPK* signaling may be a promising therapeutic target for the treatment of neuropathic pain. Further, Ma and Quirion [64] reviewed the literature, and suggested that efforts in suppressing multiple pain-related genes involved in neuropathic pain might target the *ERK*/*MAPK* pathway. While our study did not focus on neuropathic pain, among cancer patients, neuropathic pain is a debilitating sequela of malignancy and its treatment. To our knowledge, this study is the first to show the importance of these genes in studies of pain severity among cancer patients.

Other genes that showed potential association with cancer pain in HNSCC patients included *PIK3C2G* (rs10770367, *p* value = 1.10 × 10^−3^), *TCRA* (rs6572493, *p* value = 2.84 × 10^−3^), *PDGFC* (rs6845322, *p* value = 4.88 × 10^−3^), and *CD247* (rs2995082, *p* value = 7.79 × 10^-3^).

Although *PIK3C2G* has been implicated in cancer development [47, 65–67], the specific genetic variation *PIK3C2G* rs10770367, to our knowledge, had not been associated with any health risk prior to this study. Genes *TCRA* and *CD247*, both of which have at least 15 connections in the cancer pain network (Table 3), encode proteins that are essential to the assembly of the T-cell receptor-CD3 complex at the plasma membrane. The protein encoded by *CD247* is the T-cell receptor zeta; while *TCRA* gene encodes T cell receptor alpha locus receptor [68]. The T-cell receptor recognizes a specific antigen on the surface of other cells; while CD3 proteins are involved in signal transduction [69]. Our study showed that *CD247* rs2995082 and *TCRA* rs6572493 SNPs are both important to pre-treatment pain in HNSCC patients. *TCRA* rs6572493 has not yet been associated with any health risks, but *CD247* rs2995082 has been associated with celiac disease [70] and rheumatoid arthritis [71, 72].

*PDGCF-C* is a gene that encodes platelet-derived growth factor C protein. Members of the *PDGF* family are mitogens for cells of mesenchymal origin [73] and are regulators of cell migration, transformation, survival and apoptosis [65]. To our knowledge, *PDGFC* rs6845322 has not been associated with any health risk to date.

*MAPK1* rs8136867, *PIK3C2G* rs10770367, *TCRA* rs6572493, *PDGFC* rs6845322, and *CD247* rs2995082 are all located in introns [74], the non-coded sequence, of their respective genes. Introns, which are usually present in most eukaryotic genes, are removed by splicing such that the mutations in this sequence were usually thought not to alter the expressed proteins. However, recent evidence has suggested that genomic variants in the noncoding sequences (introns) can lead to deleterious gene transcript variants [75] or to alterations in gene expression levels [76] that can lead to disease or increased risk of disease. For instance, intron variants of the *p53* gene were associated with ovarian cancer risk [77], intronic SNP rs8048002 in the *MHC* class II transactivation gene (*MHC2TA*) was associated with increased risk of inflammatory disease [78], and intronic SNP rs9282860 in serine-threonine kinase 11 is a genetic risk factor in women with multiple sclerosis [79].

Among the limitations of this study is that the sizes of the networks reflect the amount of literature available on the focus genes. Also, edges are simplified in that IPA designates only a single edge between each pair of molecules in a network regardless of the number of interactions the two molecules share. Furthermore, the identified association between *MAPK1/ERK2* should be viewed as preliminary and exploratory. Multiple comparison adjustment was not performed in this analysis, and none of the associations reported would be statistically significant if such adjustments were performed. However, as the analysis in this study was considered as preliminary and exploratory, the multiple comparison adjustments are usually not required [80]. Despite these limitations however, the present study identified novel, potentially biologically meaningful candidate genes associated with cancer pain in HNSCC patients. These genes, though requiring further validation in future studies using independent data as well as other cancer sites, may allow researchers to not only identify a subgroup of the patient population and higher susceptibility for cancer associated pain and symptoms, but may also provide insight into the etiology of cancer associated pain. This in turn can be used to inform clinical decision making and help develop targeted treatment strategies for this subgroup.

## Conclusions

In conclusion, IPA is able to use large-scale information to produce comprehensive networks of genes and underlying biological pathways implicated in a phenotype. Future studies should aim to target these molecules and pathways while also minimizing adverse effects due to a lack of specificity.

### Availability of data and materials

The data bases used for the network generation of this article are available in the Ingenuity Knowledge Base by Ingenuity Pathway Analysis (IPA; Ingenuity® Systems, www.ingenuity.com). The list of articles and genes identified through the literature search are provided in Additional file 1. The data used for the association analysis of this article is from a study of squamous cell carcinoma of the head and neck conducted at The University of Texas MD Anderson Cancer Center. The list of all 800 SNPs used from this study is provided in Additional file 2. The data supporting the results of this article are included within the article and its additional files: Additional files 1 and 2 which are referenced in the main text.

## Additional files

Additional file 1:
**Genetic association studies for pain in cancer patients, sorted by publication year and name of first author, obtained through literature review.** (DOCX 87 kb) Additional file 2:
**Information of the 800 SNPs within the 82 IPA-selected genes from the Illumina HumanOmniExpress-12v1 platform.** (DOCX 72 kb)
