# Supplementary material for: MAPK1/ERK2 as novel target genes for pain in head and neck cancer patients
Source: BMC Genet. 2016 Feb 13;17:40. doi: 10.1186/s12863-016-0348-7 (PMC4752805; doi:10.1186/s12863-016-0348-7)
Supplement: Additional file 1: — Genetic association studies for pain in cancer patients, sorted by publication year and name of first author, obtained through literature review. (DOCX 87 kb) [file 12863_2016_348_MOESM1_ESM.docx]

**Table S1. Genetic association studies for pain in cancer patients, sorted by publication year and name of first author, obtained through literature review.**

| **Year** | **First author** | **Ethnicity** | **Cancer type** | **Sample size** | **Phenotype** | **Significant genes** |
| --- | --- | --- | --- | --- | --- | --- |
|  |  |  |  |  |  |  |
| 2004 | Klepstad P[^3^](#_ENREF_3) | W | N/A | 207 | Average pain during the last 24 hours (brief pain inventory) | OPRM1 |
| 2005 | Rakvåg TT[^40^](#_ENREF_40) | W | N/A | 207 | Average pain during the last 24 hours (brief pain inventory) | None |
| 2006 | Chou WY[^81^](#_ENREF_81) | A | N/A | 80 | postoperative resting pain score (visual analog scale) | None |
| 2006 | Coulbault L[^82^](#_ENREF_82) | W | N/A | 74 | Pain level (visual analogue scale) during the first 24 hours after surgery | None |
| 2006 | Wang G[^83^](#_ENREF_83) | A | Gastric | 63 | Pain score (visual analogue scale) before operation | None |
| 2007 | Reyes-Gibby CC[^9^](#_ENREF_9) | W, AA, H | Lung | 606 | Pain severity before therapy (11 point numerical scale) | IL8 |
| 2007 | Reyes-Gibby CC[^38^](#_ENREF_38) | W | N/A | 207 | Average pain during the last 24 hours (brief pain inventory) | None |
| 2008 | Campa D[^4^](#_ENREF_4) | W | N/A | 137 | Decrease in pain (numerical rating scale) after 1st week of treatment | ABCB1/MDR1, OPRM1 |
| 2008 | Hayashida M[^84^](#_ENREF_84) | A | Stomach, colon, liver, pancreas | 138 | Pain intensity (5 point numerical rating scale) at rest during the first 24 hour postoperative period | None |
| 2008 | Rakvåg TT[^41^](#_ENREF_41) | W | N/A | 197 | Average pain during the last 24 hours (brief pain inventory) | None |
| 2008 | Reyes-Gibby CC[^10^](#_ENREF_10) | W | Lung | 140 | Pain severity before therapy (11 point numerical scale) | TNFa, IL6 |
| 2008 | Ross JR[^85^](#_ENREF_85) | W | N/A | 228 | Average pain during the last 24 hours (brief pain inventory) | None |
| 2009 | Reyes-Gibby CC[^18^](#_ENREF_18) | W | Lung | 667 | Pain severity before therapy (11 point numerical scale) | PTGS2, TNFa, NFKBIA |
| 2009 | Reyes-Gibby CC[^12^](#_ENREF_12) | W | Pancreatic | 128 | Pain severity before therapy (11 point numerical scale) | IL8 |
| 2010 | Ingle JN[^76^](#_ENREF_76) | W | Breast | 878 | Aromatase inhibitor-associated musculoskeletal adverse events | TCL1A |
| 2010 | Lötsch J[^77^](#_ENREF_77) | W | N/A | 251 | Time interval between cancer diagnosis and opioid therapy initiation | GCH1 |
| 2010 | Nissenbaum J[^78^](#_ENREF_78) | W | Breast | 549 | Pain related to breast surgery during the course of the last year (yes or no) | CACNG2 |
| 2010 | Rausch SM[^13^](#_ENREF_13) | W | Lung | 1149 | Bodily pain severity (short-form general health survey-8) and Lung cancer symptom scale pain | IL-10, IL1-RN |
| 2011 | Galvan A[^39^](#_ENREF_39) | W | N/A | 1008 | Pain relief measured using an 11-point numerical rating scale (0% to 100%) | SPON1, RHBDF2, ZNF235 |
| 2011 | Hickey OT[^7^](#_ENREF_7) | W | Breast | 42 | Persistent postsurgical pain (yes or no) | COMT |
| 2011 | Mao JJ[^74^](#_ENREF_74) | W | Breast | 390 | Aromatase inhibitor-associated arthralgia (yes or no) | CYP19A1 |
| 2012 | Droney JM[^5^](#_ENREF_5) | W | N/A | 264 | Principal component variables based on subjective pain (brief pain inventory) and morphine side-effect scores | OPRK1, OPRM1 |
| 2012 | Fernández-de-las-Peñas C[^8^](#_ENREF_8) | W | Breast | 128 | Postmastectomy pain (visual analog scale) and pressure pain hypersensitivity (pressure pain thresholds) | COMT |
| 2012 | Fladvad T[^86^](#_ENREF_86) | W | Urological, lung, breast prostate gastrointestinal female reproductive organs hematological | 2201 | Average pain intensity, pain right now, worst pain intensity, pain at its least, pain relief pain interference | None |
| 2012 | Illi J[^14^](#_ENREF_14) | W, A, AA, H | Breast, prostate, lung, brain | 254 | Symptom cluster of pain, fatigue, sleep disturbance and depression | IL4 |
| 2012 | McCann B[^15^](#_ENREF_15) | W, A, AA, H | Breast | 398 | Breast pain (yes or no, breast symptom questionnaires) before surgery | IL1R1, IL13 |
| 2012 | Ochroch EA[^6^](#_ENREF_6) | W, AA, A | Lung | 90 | Postoperative day 3 pain | COX1, OPRM1 |
| 2012 | Rausch SM[^73^](#_ENREF_73) | W | Lung | 1149 | Bodily pain severity (short-form general health survey-8) | PTGS2, LTA |
| 2012 | Sloan JA[^79^](#_ENREF_79) | W | Lung | 1299 | Pain as a symptom measure (Lung cancer symptom scale and numeric linear analog self-assessment measures) | ABCC2, ABCC4 |
| 2013 | Garcia-Giralt N[^75^](#_ENREF_75) | W | Breast | 343 | Aromatase inhibitor-related arthralgia. Absolute increase in pain (visual analogic scale) at 3 and 12 months compared with that at baseline | CYP17A1, VDR, CYP27B1, CYP19A1 |
| 2013 | Gong XD[^87^](#_ENREF_87) | A | N/A | 112 | Visual analogic scale for pain before administration of opioids and after the 24 hours administration | None |
| 2013 | Kambur O[^42^](#_ENREF_42) | W | Breast | 1000 | Contact heat pain and cold pain | COMT |
| 2013 | Reyes-Gibby CC[^19^](#_ENREF_19) | W | Lung | 599 | Severity of symptom clusters of pain, depress and fatigue | ENOS, IL1B, TNFR2, PTGS2, IL10RB |
| 2013 | Reyes-Gibby CC[^16^](#_ENREF_16) | W | Lung | 599 | Pain severity before therapy (11 point numerical scale) | IL8 |
| 2014 | Stephens K[^17^](#_ENREF_17) | W, A, AA, H | Breast | 172 | Severe persistent breast pain | IFNG1, IL1R1, IL1R2, IL4, IL10, IL13, NFKB1 |
| 2014 | Wang K[^80^](#_ENREF_80) | A | Pancreatic | 30 | Abdominal pain sensation | GFRa-2 |

W: White; A: Asian; AA: African American; H: Hispanic
