# Supplementary material for: MAPK1/ERK2 as novel target genes for pain in head and neck cancer patients
Source: BMC Genet. 2016 Feb 13;17:40. doi: 10.1186/s12863-016-0348-7 (PMC4752805; doi:10.1186/s12863-016-0348-7)
Supplement: Additional file 2: — Information of the 800 SNPs within the 82 IPA-selected genes from the Illumina HumanOmniExpress-12v1 platform. (DOCX 72 kb) [file 12863_2016_348_MOESM2_ESM.docx]

**Table S2. Information of the 800 SNPs within the 82 IPA-selected genes from the Illumina HumanOmniExpress-12v1 platform^*^. The molecules in boldface are focus genes.**

| **IPA Symbols** | **Genes** | **rs#** | **Relationship to genes** |
| --- | --- | --- | --- |
|  |  |  |  |
| **TNF** | TNF | rs1800630 | downstream-variant-500B,upstream-variant-2KB^ǂ^ |
|  |  | rs1800629 | upstream-variant-2KB |
|  |  | rs3093662 | intron-variant |
| **IFNG** | IFNG | rs2069727 | downstream-variant-500B |
|  |  | rs2069718 | intron-variant |
| **IL1B** | IL1B | rs1143634 | synonymous-codon |
|  |  | rs1143633 | intron-variant |
|  |  | rs1143627 | upstream-variant-2KB |
|  |  | rs16944 | upstream-variant-2KB |
| **CXCL8** | CXCL8 | rs2227543 | intron-variant |
| **IL6** | IL6 | rs2069827 | nc-transcript-variant,upstream-variant-2KB,utr-variant-5-prime^ǂ^ |
|  |  | rs1800797 | intron-variant,nc-transcript-variant,upstream-variant-2KB^ǂ^ |
|  |  | rs2069832 | intron-variant,upstream-variant-2KB^ǂ^ |
|  |  | rs2069835 | intron-variant,upstream-variant-2KB^ǂ^ |
|  |  | rs2069837 | intron-variant,upstream-variant-2KB^ǂ^ |
|  |  | rs1474347 | intron-variant,upstream-variant-2KB^ǂ^ |
|  |  | rs1554606 | intron-variant,upstream-variant-2KB^ǂ^ |
| Lh | CGA | rs9359730 | unknown |
|  |  | rs1055409 | utr-variant-3-prime |
|  |  | rs9342104 | intron-variant |
|  | LHB | rs753307 | intron-variant |
|  |  | rs1056917 | downstream-variant-500B,synonymous-codon^ǂ^ |
| FSH | CGA | rs9359730 | unknown |
|  |  | rs1055409 | utr-variant-3-prime |
|  |  | rs9342104 | intron-variant |
|  | FSHB | rs12295914 | unknown |
| NFkB (complex) | NFKB1 | rs17032705 | intron-variant |
|  |  | rs1599961 | intron-variant |
|  |  | rs1598856 | intron-variant,upstream-variant-2KB^ǂ^ |
|  |  | rs230535 | intron-variant |
|  |  | rs13117745 | intron-variant |
|  |  | rs4648090 | intron-variant |
|  |  | rs230547 | intron-variant,nc-transcript-variant^ǂ^ |
|  |  | rs4648135 | intron-variant,nc-transcript-variant^ǂ^ |
|  |  | rs1609798 | intron-variant,upstream-variant-2KB^ǂ^ |
|  | NFKB2 | rs7897947 | intron-variant |
|  |  | rs11574849 | intron-variant |
|  |  | rs1056890 | downstream-variant-500B,nc-transcript-variant,utr-variant-3-prime^ǂ^ |
|  | REL | rs6545835 | intron-variant |
|  |  | rs12713428 | intron-variant |
|  |  | rs34023434 | intron-variant |
|  |  | rs10193964 | intron-variant,upstream-variant-2KB^ǂ^ |
|  |  | rs1429265 | utr-variant-3-prime |
|  |  | rs3732179 | utr-variant-3-prime |
|  | RELA | rs11227247 | intron-variant |
|  |  | rs11820062 | intron-variant,upstream-variant-2KB^ǂ^ |
|  |  | rs7101916 | intron-variant,upstream-variant-2KB^ǂ^ |
|  | RELB | rs2288918 | intron-variant |
| **IL10** | IL10 | rs3024498 | utr-variant-3-prime |
|  |  | rs3024496 | utr-variant-3-prime |
|  |  | rs3021094 | intron-variant |
|  |  | rs1800896 | intron-variant,upstream-variant-2KB^ǂ^ |
| P38 MAPK | MAPK1 | rs9610271 | unknown |
|  |  | rs9340 | utr-variant-3-prime |
|  |  | rs3810610 | utr-variant-3-prime |
|  |  | rs2298432 | downstream-variant-500B,intron-variant^ǂ^ |
|  |  | rs2006893 | intron-variant |
|  |  | rs9607272 | intron-variant |
|  |  | rs5999521 | intron-variant |
|  |  | rs7290469 | intron-variant |
|  |  | rs9610375 | intron-variant |
|  |  | rs17759796 | intron-variant |
|  |  | rs9610417 | intron-variant |
|  |  | rs8136867 | intron-variant |
|  | MAPK11 | rs2076139 | nc-transcript-variant,synonymous-codon^ǂ^ |
|  |  | rs742186 | intron-variant,upstream-variant-2KB^ǂ^ |
|  | MAPK12 | rs1555048 | synonymous-codon |
|  |  | rs1129880 | synonymous-codon |
|  |  | rs742184 | intron-variant |
|  | MAPK13 | rs1059227 | nc-transcript-variant,synonymous-codon^ǂ^ |
|  |  | rs2071864 | intron-variant |
|  |  | rs2071863 | nc-transcript-variant,utr-variant-3-prime^ǂ^ |
|  | MAPK14 | rs3761979 | upstream-variant-2KB |
|  |  | rs851023 | intron-variant |
|  |  | rs3804454 | intron-variant |
|  |  | rs12199654 | intron-variant |
|  |  | rs851019 | intron-variant |
|  |  | rs2237093 | intron-variant |
|  |  | rs851006 | intron-variant |
|  |  | rs2815806 | downstream-variant-500B,intron-variant^ǂ^ |
|  |  | rs7760405 | intron-variant |
|  |  | rs9470219 | intron-variant |
|  |  | rs6457878 | utr-variant-3-prime |
|  |  | rs3804452 | utr-variant-3-prime |
|  |  | rs3804451 | utr-variant-3-prime |
| ERK1/2 | MAPK1 | rs9610271 | unknown |
|  |  | rs9340 | utr-variant-3-prime |
|  |  | rs3810610 | utr-variant-3-prime |
|  |  | rs2298432 | downstream-variant-500B,intron-variant^ǂ^ |
|  |  | rs2006893 | intron-variant |
|  |  | rs9607272 | intron-variant |
|  |  | rs5999521 | intron-variant |
|  |  | rs7290469 | intron-variant |
|  |  | rs9610375 | intron-variant |
|  |  | rs17759796 | intron-variant |
|  |  | rs9610417 | intron-variant |
|  |  | rs8136867 | intron-variant |
|  | MAPK3 | rs11865086 | intron-variant |
| **PTGS2** | PTGS2 | rs689467 | utr-variant-3-prime |
|  |  | rs2206593 | utr-variant-3-prime |
|  |  | rs5275 | utr-variant-3-prime |
| **IL4** | IL4 | rs2243248 | upstream-variant-2KB |
|  |  | rs2243250 | upstream-variant-2KB |
|  |  | rs2070874 | utr-variant-5-prime |
|  |  | rs2243268 | intron-variant |
|  |  | rs2243290 | intron-variant,nc-transcript-variant^ǂ^ |
| **IL1RN** | IL1RN | rs1688072 | intron-variant |
|  |  | rs315931 | intron-variant |
|  |  | rs17042917 | utr-variant-5-prime |
|  |  | rs315919 | intron-variant |
|  |  | rs4251985 | intron-variant |
|  |  | rs3213448 | intron-variant |
|  |  | rs419598 | synonymous-codon |
|  |  | rs423904 | intron-variant |
|  |  | rs431726 | intron-variant |
|  |  | rs3087266 | intron-variant |
|  |  | rs315952 | synonymous-codon |
|  |  | rs9005 | utr-variant-3-prime |
| CD3 | CD3G | rs2071381 | upstream-variant-2KB |
|  |  | rs3212262 | intron-variant |
|  |  | rs1561966 | intron-variant,stop-gained^ǂ^ |
|  | CD3D | rs2276424 | intron-variant |
|  |  | rs3181261 | intron-variant |
|  |  | rs2239695 | upstream-variant-2KB |
|  | CD3E | rs3825051 | upstream-variant-2KB |
|  |  | rs2231440 | utr-variant-5-prime |
|  |  | rs4606515 | synonymous-codon |
|  |  | rs2277289 | intron-variant |
|  |  | rs1945764 | intron-variant |
|  | CD247 | rs870873 | downstream-variant-500B |
|  |  | rs870875 | downstream-variant-500B |
|  |  | rs953809 | intron-variant |
|  |  | rs1723023 | intron-variant |
|  |  | rs2995082 | intron-variant |
|  |  | rs2258497 | intron-variant |
|  |  | rs12141641 | intron-variant |
|  |  | rs864537 | intron-variant |
|  |  | rs1554669 | intron-variant |
|  |  | rs7523907 | intron-variant,nc-transcript-variant^ǂ^ |
|  |  | rs2988276 | intron-variant |
|  |  | rs10918693 | intron-variant |
|  |  | rs1723015 | intron-variant |
|  |  | rs1723016 | intron-variant |
|  |  | rs10918695 | intron-variant |
|  |  | rs2480678 | intron-variant |
|  |  | rs2995093 | intron-variant |
|  |  | rs7518703 | intron-variant |
|  |  | rs1214609 | intron-variant |
|  |  | rs12737372 | intron-variant |
|  |  | rs2949655 | intron-variant |
|  |  | rs16859085 | intron-variant |
|  |  | rs858553 | intron-variant |
|  |  | rs863455 | intron-variant |
|  |  | rs6670426 | intron-variant |
|  |  | rs704852 | intron-variant |
|  |  | rs858543 | intron-variant,nc-transcript-variant^ǂ^ |
|  |  | rs704853 | intron-variant,nc-transcript-variant^ǂ^ |
|  |  | rs1799704 | intron-variant |
|  |  | rs2982484 | intron-variant |
|  |  | rs858550 | intron-variant |
|  |  | rs12095738 | intron-variant |
|  |  | rs3820390 | upstream-variant-2KB |
| Vegf | VEGFA | rs25648 | synonymous-codon,upstream-variant-2KB,utr-variant-5-prime^ǂ^ |
|  |  | rs833068 | intron-variant |
|  |  | rs833069 | intron-variant |
|  |  | rs3025010 | intron-variant |
|  |  | rs3025035 | intron-variant |
|  |  | rs3025053 | utr-variant-3-prime |
|  | VEGFB | rs11603042 | intron-variant |
|  | VEGFC | rs1485762 | intron-variant |
|  |  | rs7664413 | intron-variant |
|  |  | rs1485766 | intron-variant |
|  |  | rs3775202 | intron-variant |
|  |  | rs3775198 | intron-variant |
|  |  | rs3775195 | intron-variant |
|  |  | rs6820170 | intron-variant |
|  |  | rs475106 | intron-variant |
|  |  | rs10012721 | intron-variant |
|  |  | rs13122901 | intron-variant |
|  |  | rs4557213 | intron-variant |
|  | FIGF ^Ɨ^ | - | - |
|  | PDGFC | rs1425486 | nc-transcript-variant,utr-variant-3-prime^ǂ^ |
|  |  | rs983473 | intron-variant |
|  |  | rs6834932 | intron-variant |
|  |  | rs4691381 | intron-variant |
|  |  | rs10517653 | intron-variant |
|  |  | rs10033375 | intron-variant |
|  |  | rs10008978 | intron-variant |
|  |  | rs2974254 | intron-variant |
|  |  | rs7682857 | intron-variant |
|  |  | rs2911941 | intron-variant |
|  |  | rs6811964 | intron-variant |
|  |  | rs10517657 | intron-variant |
|  |  | rs342317 | intron-variant |
|  |  | rs1469243 | intron-variant |
|  |  | rs6812101 | intron-variant |
|  |  | rs17231377 | intron-variant |
|  |  | rs4691383 | intron-variant |
|  |  | rs17231573 | intron-variant |
|  |  | rs10517660 | intron-variant |
|  |  | rs10010472 | intron-variant |
|  |  | rs6845322 | intron-variant |
|  | PGF | rs2268613 | downstream-variant-500B |
|  |  | rs8185 | utr-variant-3-prime |
|  | PROK1 | rs1857512 | upstream-variant-2KB |
|  |  | rs4839391 | intron-variant |
|  |  | rs3795828 | intron-variant |
|  |  | rs17628304 | intron-variant |
|  |  | rs1416815 | intron-variant |
|  |  | rs7514102 | missense |
| **IL13** | IL13 | rs1881457 | upstream-variant-2KB |
|  |  | rs1295686 | intron-variant |
|  |  | rs20541 | missense |
|  |  | rs1295685 | utr-variant-3-prime |
|  |  | rs848 | utr-variant-3-prime |
| PI3K (complex) | ATM | rs4987876 | intron-variant,upstream-variant-2KB^ǂ^ |
|  |  | rs228591 | intron-variant |
|  |  | rs11212570 | intron-variant |
|  |  | rs609261 | intron-variant,upstream-variant-2KB^ǂ^ |
|  |  | rs619972 | intron-variant |
|  |  | rs599558 | intron-variant |
|  |  | rs425538 | downstream-variant-500B,intron-variant^ǂ^ |
|  |  | rs664143 | intron-variant |
|  |  | rs4585 | intron-variant,utr-variant-3-prime^ǂ^ |
|  | PIK3C2A | rs16933823 | upstream-variant-2KB |
|  |  | rs12577388 | upstream-variant-2KB |
|  |  | rs10832733 | utr-variant-3-prime |
|  |  | rs11604561 | missense |
|  |  | rs11603765 | intron-variant |
|  |  | rs214936 | intron-variant,synonymous-codon^ǂ^ |
|  |  | rs214933 | intron-variant |
|  | PIK3C2B | rs4951373 | utr-variant-3-prime |
|  |  | rs17406271 | intron-variant |
|  |  | rs17334387 | nc-transcript-variant,synonymous-codon^ǂ^ |
|  |  | rs2271421 | intron-variant |
|  |  | rs12031854 | intron-variant |
|  |  | rs2292459 | intron-variant |
|  |  | rs3106366 | intron-variant |
|  |  | rs2271427 | intron-variant |
|  |  | rs1008833 | intron-variant |
|  |  | rs2999484 | intron-variant |
|  |  | rs16853770 | intron-variant |
|  |  | rs2271415 | intron-variant |
|  |  | rs7556371 | intron-variant |
|  |  | rs11240751 | intron-variant |
|  | PIK3C2G | rs10770333 | intron-variant |
|  |  | rs10770343 | intron-variant,upstream-variant-2KB^ǂ^ |
|  |  | rs4385947 | intron-variant |
|  |  | rs11043991 | intron-variant |
|  |  | rs17409120 | intron-variant |
|  |  | rs11044004 | missense,nc-transcript-variant,utr-variant-5-prime^ǂ^ |
|  |  | rs7133666 | missense,nc-transcript-variant^ǂ^ |
|  |  | rs10841010 | intron-variant |
|  |  | rs12582971 | intron-variant |
|  |  | rs9300118 | intron-variant |
|  |  | rs4387408 | intron-variant |
|  |  | rs4609650 | intron-variant |
|  |  | rs16914120 | intron-variant |
|  |  | rs10505810 | intron-variant |
|  |  | rs12309567 | intron-variant |
|  |  | rs12582985 | intron-variant |
|  |  | rs12426122 | intron-variant |
|  |  | rs12297325 | intron-variant |
|  |  | rs4764395 | intron-variant |
|  |  | rs12371012 | intron-variant |
|  |  | rs11044078 | intron-variant |
|  |  | rs12822135 | intron-variant |
|  |  | rs11044103 | intron-variant |
|  |  | rs12427286 | intron-variant |
|  |  | rs9634063 | intron-variant |
|  |  | rs1447406 | intron-variant |
|  |  | rs12371624 | intron-variant |
|  |  | rs7314398 | intron-variant |
|  |  | rs1868064 | intron-variant |
|  |  | rs1447408 | intron-variant |
|  |  | rs10743273 | intron-variant |
|  |  | rs1374670 | intron-variant |
|  |  | rs12312266 | intron-variant,missense^ǂ^ |
|  |  | rs2290044 | intron-variant |
|  |  | rs2305220 | intron-variant |
|  |  | rs10770367 | intron-variant |
|  |  | rs11044165 | intron-variant |
|  |  | rs7969452 | intron-variant |
|  |  | rs11044171 | intron-variant |
|  |  | rs1992838 | intron-variant |
|  |  | rs10841036 | intron-variant |
|  |  | rs12813137 | intron-variant |
|  |  | rs12827287 | intron-variant |
|  |  | rs1816971 | intron-variant |
|  |  | rs7964182 | intron-variant |
|  |  | rs11837598 | intron-variant |
|  |  | rs12825580 | intron-variant |
|  |  | rs4764409 | intron-variant |
|  |  | rs4763508 | intron-variant |
|  |  | rs518345 | intron-variant |
|  |  | rs11044235 | downstream-variant-500B,intron-variant^ǂ^ |
|  | PIK3C3 | rs3813065 | upstream-variant-2KB |
|  |  | rs9956832 | intron-variant |
|  |  | rs11082257 | intron-variant |
|  |  | rs1941526 | intron-variant |
|  |  | rs504302 | nc-transcript-variant,utr-variant-3-prime^ǂ^ |
|  | PIK3CA | rs11919383 | intron-variant |
|  |  | rs11706842 | intron-variant |
|  |  | rs7635669 | intron-variant |
|  |  | rs7651265 | intron-variant |
|  |  | rs6443624 | intron-variant |
|  |  | rs2677760 | intron-variant |
|  |  | rs9866361 | intron-variant |
|  |  | rs2677770 | intron-variant |
|  |  | rs2677764 | intron-variant |
|  |  | rs4854957 | intron-variant |
|  |  | rs13320527 | intron-variant |
|  |  | rs1607237 | intron-variant |
|  |  | rs9838117 | downstream-variant-500B,utr-variant-3-prime^ǂ^ |
|  | PIK3CB | rs559160 | intron-variant |
|  |  | rs524164 | intron-variant |
|  |  | rs361059 | intron-variant |
|  |  | rs10513055 | intron-variant |
|  |  | rs500687 | intron-variant |
|  | PIK3CD | rs7518793 | intron-variant,upstream-variant-2KB^ǂ^ |
|  |  | rs4240895 | intron-variant,nc-transcript-variant^ǂ^ |
|  |  | rs4240896 | intron-variant,nc-transcript-variant^ǂ^ |
|  |  | rs12039250 | intron-variant |
|  |  | rs4333853 | intron-variant |
|  |  | rs6677649 | intron-variant |
|  |  | rs6669509 | intron-variant,upstream-variant-2KB^ǂ^ |
|  |  | rs12566637 | intron-variant |
|  |  | rs12075554 | intron-variant |
|  |  | rs6541017 | intron-variant |
|  |  | rs3934934 | intron-variant |
|  |  | rs9430220 | intron-variant |
|  |  | rs11589267 | intron-variant |
|  |  | rs10864435 | intron-variant |
|  |  | rs1135426 | nc-transcript-variant,utr-variant-3-prime^ǂ^ |
|  |  | rs1135427 | nc-transcript-variant,utr-variant-3-prime^ǂ^ |
|  |  | rs7526958 | utr-variant-3-prime |
|  | PIK3CG | rs4730204 | upstream-variant-2KB |
|  |  | rs4727666 | upstream-variant-2KB |
|  |  | rs4460309 | intron-variant |
|  |  | rs12536620 | intron-variant |
|  |  | rs12667819 | utr-variant-3-prime |
|  |  | rs11766675 | utr-variant-3-prime |
|  | PIK3R1 | rs706713 | synonymous-codon |
|  |  | rs13173003 | intron-variant |
|  |  | rs7713645 | intron-variant |
|  |  | rs7709243 | intron-variant,upstream-variant-2KB^ǂ^ |
|  |  | rs12652661 | intron-variant,upstream-variant-2KB^ǂ^ |
|  |  | rs251406 | intron-variant |
|  |  | rs173704 | intron-variant |
|  |  | rs173702 | intron-variant |
|  |  | rs4122269 | intron-variant |
|  |  | rs1823023 | intron-variant,upstream-variant-2KB^ǂ^ |
|  |  | rs173703 | intron-variant,upstream-variant-2KB^ǂ^ |
|  |  | rs6893676 | intron-variant |
|  |  | rs34303 | intron-variant |
|  |  | rs863818 | intron-variant |
|  |  | rs34309 | intron-variant |
|  |  | rs2302975 | intron-variant |
|  |  | rs2302976 | intron-variant |
|  |  | rs3730082 | intron-variant |
|  |  | rs6876003 | intron-variant |
|  |  | rs7716675 | intron-variant |
|  |  | rs12656176 | intron-variant |
|  |  | rs3815701 | intron-variant |
|  |  | rs34306 | intron-variant |
|  |  | rs1550805 | intron-variant,upstream-variant-2KB^ǂ^ |
|  |  | rs3730089 | missense,upstream-variant-2KB^ǂ^ |
|  |  | rs1043526 | utr-variant-3-prime |
|  |  | rs3756668 | utr-variant-3-prime |
|  | PIK3R2 | rs2006885 | intron-variant |
|  |  | rs3730179 | intron-variant |
|  | PIK3R3 | rs785468 | intron-variant,synonymous-codon^ǂ^ |
|  |  | rs1085243 | intron-variant |
|  |  | rs785520 | intron-variant |
|  |  | rs1707303 | intron-variant,upstream-variant-2KB,utr-variant-5-prime^ǂ^ |
|  |  | rs1707302 | intron-variant |
|  |  | rs785475 | intron-variant |
|  |  | rs7512966 | intron-variant |
|  |  | rs3855959 | intron-variant |
|  |  | rs3845301 | intron-variant |
|  | PIK3R4 | rs10934954 | intron-variant |
|  |  | rs2170990 | synonymous-codon |
|  |  | rs2293179 | utr-variant-5-prime |
|  | PIK3R5 | rs4791764 | intron-variant |
|  |  | rs4791765 | intron-variant |
|  |  | rs373495 | intron-variant |
|  |  | rs374864 | intron-variant |
|  |  | rs9915880 | intron-variant,synonymous-codon,utr-variant-5-prime^ǂ^ |
|  |  | rs381309 | intron-variant,synonymous-codon,utr-variant-5-prime^ǂ^ |
|  |  | rs394811 | missense,synonymous-codon,utr-variant-5-prime^ǂ^ |
|  |  | rs411268 | intron-variant |
|  |  | rs366259 | intron-variant |
|  |  | rs427554 | intron-variant |
|  |  | rs726680 | intron-variant |
|  |  | rs726679 | intron-variant |
|  |  | rs9895992 | intron-variant |
|  |  | rs12453818 | intron-variant |
|  |  | rs4791769 | intron-variant |
|  |  | rs10491087 | intron-variant |
|  |  | rs11657742 | intron-variant |
|  |  | rs714407 | intron-variant |
|  | PIK3R6 | rs9912143 | intron-variant,missense,nc-transcript-variant,utr-variant-3-prime^ǂ^ |
|  |  | rs195770 | intron-variant |
|  |  | rs2074258 | intron-variant |
|  |  | rs12944446 | intron-variant |
|  |  | rs12944785 | intron-variant |
|  |  | rs8064777 | intron-variant |
|  |  | rs9910718 | intron-variant,upstream-variant-2KB^ǂ^ |
|  |  | rs9903136 | intron-variant |
|  |  | rs443141 | intron-variant |
|  |  | rs8067644 | intron-variant |
| IL12 (complex) | IL12A | rs2243115 | intron-variant,upstream-variant-2KB^ǂ^ |
|  |  | rs2243123 | intron-variant |
|  |  | rs568408 | intron-variant,utr-variant-3-prime^ǂ^ |
|  | IL12B | rs3212227 | utr-variant-3-prime |
|  |  | rs11574790 | intron-variant |
|  |  | rs2853694 | intron-variant |
|  |  | rs3213094 | intron-variant |
|  |  | rs2569254 | intron-variant |
|  |  | rs1003199 | intron-variant |
|  |  | rs730691 | intron-variant |
| **NFKB1** | NFKB1 | rs17032705 | intron-variant |
|  |  | rs1599961 | intron-variant |
|  |  | rs1598856 | intron-variant,upstream-variant-2KB^ǂ^ |
|  |  | rs230535 | intron-variant |
|  |  | rs13117745 | intron-variant |
|  |  | rs4648090 | intron-variant |
|  |  | rs230547 | intron-variant,nc-transcript-variant^ǂ^ |
|  |  | rs4648135 | intron-variant,nc-transcript-variant^ǂ^ |
|  |  | rs1609798 | intron-variant,upstream-variant-2KB^ǂ^ |
| TCR | CD3G | rs2071381 | upstream-variant-2KB |
|  |  | rs3212262 | intron-variant |
|  |  | rs1561966 | intron-variant,stop-gained^ǂ^ |
|  | CD3D | rs2276424 | intron-variant |
|  |  | rs3181261 | intron-variant |
|  |  | rs2239695 | upstream-variant-2KB |
|  | CD3E | rs3825051 | upstream-variant-2KB |
|  |  | rs2231440 | utr-variant-5-prime |
|  |  | rs4606515 | synonymous-codon |
|  |  | rs2277289 | intron-variant |
|  |  | rs1945764 | intron-variant |
|  | CD247 | rs870873 | downstream-variant-500B |
|  |  | rs870875 | downstream-variant-500B |
|  |  | rs953809 | intron-variant |
|  |  | rs1723023 | intron-variant |
|  |  | rs2995082 | intron-variant |
|  |  | rs2258497 | intron-variant |
|  |  | rs12141641 | intron-variant |
|  |  | rs864537 | intron-variant |
|  |  | rs1554669 | intron-variant |
|  |  | rs7523907 | intron-variant,nc-transcript-variant^ǂ^ |
|  |  | rs2988276 | intron-variant |
|  |  | rs10918693 | intron-variant |
|  |  | rs1723015 | intron-variant |
|  |  | rs1723016 | intron-variant |
|  |  | rs10918695 | intron-variant |
|  |  | rs2480678 | intron-variant |
|  |  | rs2995093 | intron-variant |
|  |  | rs7518703 | intron-variant |
|  |  | rs1214609 | intron-variant |
|  |  | rs12737372 | intron-variant |
|  |  | rs2949655 | intron-variant |
|  |  | rs16859085 | intron-variant |
|  |  | rs858553 | intron-variant |
|  |  | rs863455 | intron-variant |
|  |  | rs6670426 | intron-variant |
|  |  | rs704852 | intron-variant |
|  |  | rs858543 | intron-variant,nc-transcript-variant^ǂ^ |
|  |  | rs704853 | intron-variant,nc-transcript-variant^ǂ^ |
|  |  | rs1799704 | intron-variant |
|  |  | rs2982484 | intron-variant |
|  |  | rs858550 | intron-variant |
|  |  | rs12095738 | intron-variant |
|  |  | rs3820390 | upstream-variant-2KB |
|  | FCER1G | rs1136224 | upstream-variant-2KB,utr-variant-3-prime^ǂ^ |
|  |  | rs11587213 | upstream-variant-2KB |
|  |  | rs12094497 | intron-variant |
|  |  | rs2070902 | intron-variant |
|  |  | rs4489574 | unknown |
|  | TCRA | rs2049786 | unknown |
|  |  | rs12434436 | unknown |
|  |  | rs2051533 | unknown |
|  |  | rs2051534 | unknown |
|  |  | rs10483253 | unknown |
|  |  | rs4982473 | unknown |
|  |  | rs4982475 | downstream-variant-500B |
|  |  | rs10146821 | missense |
|  |  | rs6571976 | unknown |
|  |  | rs2049787 | unknown |
|  |  | rs12878444 | unknown |
|  |  | rs12889057 | unknown |
|  |  | rs8021892 | unknown |
|  |  | rs10146733 | upstream-variant-2KB |
|  |  | rs10138803 | upstream-variant-2KB |
|  |  | rs2874103 | missense |
|  |  | rs970382 | missense |
|  |  | rs12892667 | unknown |
|  |  | rs6571990 | unknown |
|  |  | rs11627252 | unknown |
|  |  | rs9323020 | unknown |
|  |  | rs12882371 | unknown |
|  |  | rs8020038 | unknown |
|  |  | rs4982487 | unknown |
|  |  | rs10151114 | unknown |
|  |  | rs8013476 | unknown |
|  |  | rs7150049 | unknown |
|  |  | rs2049788 | unknown |
|  |  | rs4982495 | unknown |
|  |  | rs12435882 | unknown |
|  |  | rs4329837 | unknown |
|  |  | rs7143791 | unknown |
|  |  | rs12897392 | unknown |
|  |  | rs8021012 | unknown |
|  |  | rs7147972 | unknown |
|  |  | rs714618 | unknown |
|  |  | rs10138170 | unknown |
|  |  | rs12050401 | unknown |
|  |  | rs7155867 | unknown |
|  |  | rs2320062 | unknown |
|  |  | rs1978024 | unknown |
|  |  | rs2204955 | unknown |
|  |  | rs2320063 | unknown |
|  |  | rs2293710 | unknown |
|  |  | rs876093 | unknown |
|  |  | rs11157268 | unknown |
|  |  | rs10140834 | unknown |
|  |  | rs726976 | unknown |
|  |  | rs17182867 | unknown |
|  |  | rs2320065 | unknown |
|  |  | rs17197550 | unknown |
|  |  | rs9323066 | unknown |
|  |  | rs2874146 | unknown |
|  |  | rs733290 | unknown |
|  |  | rs733203 | unknown |
|  |  | rs4982524 | unknown |
|  |  | rs10483263 | unknown |
|  |  | rs2293728 | unknown |
|  |  | rs2293729 | unknown |
|  |  | rs7161350 | unknown |
|  |  | rs7493100 | unknown |
|  |  | rs4981390 | unknown |
|  |  | rs723879 | unknown |
|  |  | rs7157624 | unknown |
|  |  | rs2204943 | unknown |
|  |  | rs11629117 | unknown |
|  |  | rs2178779 | unknown |
|  |  | rs2242539 | unknown |
|  |  | rs17112518 | unknown |
|  |  | rs1040297 | unknown |
|  |  | rs17182972 | unknown |
|  |  | rs11624587 | unknown |
|  |  | rs2222924 | unknown |
|  |  | rs4340239 | unknown |
|  |  | rs1534813 | unknown |
|  |  | rs11620811 | unknown |
|  |  | rs11621148 | unknown |
|  |  | rs17183020 | unknown |
|  |  | rs3811327 | unknown |
|  |  | rs11852093 | unknown |
|  |  | rs11846015 | unknown |
|  |  | rs10483267 | unknown |
|  |  | rs17197676 | unknown |
|  |  | rs3827932 | unknown |
|  |  | rs3811322 | unknown |
|  |  | rs2222928 | unknown |
|  |  | rs8009990 | unknown |
|  |  | rs11850341 | unknown |
|  |  | rs8014727 | unknown |
|  |  | rs4417485 | unknown |
|  |  | rs17183069 | unknown |
|  |  | rs4981402 | unknown |
|  |  | rs7147431 | unknown |
|  |  | rs2222933 | unknown |
|  |  | rs4636818 | unknown |
|  |  | rs12887704 | unknown |
|  |  | rs2331489 | unknown |
|  |  | rs10143413 | unknown |
|  |  | rs7157434 | unknown |
|  |  | rs3811313 | unknown |
|  |  | rs2204979 | unknown |
|  |  | rs8022660 | unknown |
|  |  | rs3811302 | unknown |
|  |  | rs4982548 | unknown |
|  |  | rs7159906 | unknown |
|  |  | rs7142153 | unknown |
|  |  | rs2272549 | unknown |
|  |  | rs990962 | unknown |
|  |  | rs8022578 | unknown |
|  |  | rs4572291 | unknown |
|  |  | rs4982558 | unknown |
|  |  | rs12435990 | unknown |
|  |  | rs1040302 | unknown |
|  |  | rs2331494 | unknown |
|  |  | rs11157417 | unknown |
|  |  | rs4573856 | unknown |
|  |  | rs17197802 | unknown |
|  |  | rs2178785 | unknown |
|  |  | rs2272550 | unknown |
|  |  | rs12431484 | unknown |
|  |  | rs8015724 | unknown |
|  |  | rs7150327 | unknown |
|  |  | rs6572287 | unknown |
|  |  | rs8010032 | unknown |
|  |  | rs7143714 | unknown |
|  |  | rs4982566 | unknown |
|  |  | rs8016767 | unknown |
|  |  | rs8012078 | unknown |
|  |  | rs10872983 | unknown |
|  |  | rs2075492 | unknown |
|  |  | rs12884608 | unknown |
|  |  | rs10483268 | unknown |
|  |  | rs1894371 | unknown |
|  |  | rs12894150 | intron-variant |
|  |  | rs933193 | unknown |
|  |  | rs3811280 | unknown |
|  |  | rs7140942 | unknown |
|  |  | rs2204968 | unknown |
|  |  | rs17198033 | unknown |
|  |  | rs2075496 | unknown |
|  |  | rs10483269 | unknown |
|  |  | rs17115614 | unknown |
|  |  | rs7156103 | unknown |
|  |  | rs8022196 | unknown |
|  |  | rs12431534 | unknown |
|  |  | rs1023437 | unknown |
|  |  | rs6572331 | unknown |
|  |  | rs3811273 | unknown |
|  |  | rs4982590 | unknown |
|  |  | rs17115760 | unknown |
|  |  | rs7150691 | unknown |
|  |  | rs7155927 | unknown |
|  |  | rs7141113 | unknown |
|  |  | rs6572349 | unknown |
|  |  | rs12881142 | unknown |
|  |  | rs2331599 | unknown |
|  |  | rs8016619 | unknown |
|  |  | rs9972232 | unknown |
|  |  | rs3811272 | unknown |
|  |  | rs3811266 | unknown |
|  |  | rs17255668 | unknown |
|  |  | rs4435168 | unknown |
|  |  | rs17116039 | unknown |
|  |  | rs4981419 | unknown |
|  |  | rs10129606 | unknown |
|  |  | rs17198314 | unknown |
|  |  | rs17198328 | unknown |
|  |  | rs11624173 | unknown |
|  |  | rs9323119 | unknown |
|  |  | rs722448 | unknown |
|  |  | rs1540268 | unknown |
|  |  | rs3811259 | unknown |
|  |  | rs11845134 | unknown |
|  |  | rs10047935 | unknown |
|  |  | rs2204990 | unknown |
|  |  | rs17793809 | unknown |
|  |  | rs4981422 | unknown |
|  |  | rs10136383 | unknown |
|  |  | rs4981423 | unknown |
|  |  | rs3811256 | unknown |
|  |  | rs2032442 | unknown |
|  |  | rs2331662 | unknown |
|  |  | rs8014927 | unknown |
|  |  | rs4982619 | unknown |
|  |  | rs11157552 | intron-variant |
|  |  | rs3811244 | intron-variant |
|  |  | rs3811240 | intron-variant |
|  |  | rs1040303 | intron-variant |
|  |  | rs7150307 | intron-variant |
|  |  | rs760019 | intron-variant |
|  |  | rs3811236 | intron-variant |
|  |  | rs2301201 | intron-variant,nc-transcript-variant^ǂ^ |
|  |  | rs11846244 | intron-variant |
|  |  | rs6572449 | intron-variant,upstream-variant-2KB^ǂ^ |
|  |  | rs2141988 | upstream-variant-2KB |
|  |  | rs3811232 | unknown |
|  |  | rs10873018 | unknown |
|  |  | rs12147516 | unknown |
|  |  | rs10483273 | unknown |
|  |  | rs11848747 | unknown |
|  |  | rs11850145 | unknown |
|  |  | rs2242545 | unknown |
|  |  | rs1882704 | unknown |
|  |  | rs1882705 | downstream-variant-500B |
|  |  | rs2733777 | intron-variant,nc-transcript-variant^ǂ^ |
|  |  | rs11622344 | intron-variant,nc-transcript-variant^ǂ^ |
|  |  | rs11846670 | intron-variant |
|  |  | rs2293732 | intron-variant |
|  |  | rs1076861 | intron-variant |
|  |  | rs2254272 | intron-variant |
|  |  | rs10132733 | intron-variant |
|  |  | rs1872159 | intron-variant |
|  |  | rs227859 | intron-variant |
|  |  | rs762578 | nc-transcript-variant |
|  |  | rs227866 | upstream-variant-2KB |
|  |  | rs227867 | upstream-variant-2KB |
|  |  | rs2070982 | unknown |
|  |  | rs227869 | unknown |
|  |  | rs227870 | unknown |
|  |  | rs1483971 | unknown |
|  |  | rs2128997 | unknown |
|  |  | rs226985 | unknown |
|  |  | rs6572493 | unknown |
|  |  | rs7147589 | unknown |
|  |  | rs226996 | unknown |
|  |  | rs226998 | unknown |
|  |  | rs226999 | unknown |
|  |  | rs1483973 | unknown |
|  |  | rs1483974 | unknown |
|  |  | rs227000 | unknown |
|  |  | rs12887424 | unknown |
|  |  | rs170544 | unknown |
|  |  | rs3764159 | unknown |
|  |  | rs227028 | unknown |
|  |  | rs179106 | unknown |
|  |  | rs12587781 | unknown |
|  |  | rs227003 | unknown |
|  |  | rs1263640 | unknown |
|  |  | rs7147412 | unknown |
|  |  | rs12431397 | unknown |
|  |  | rs1263659 | unknown |
|  |  | rs438538 | unknown |
|  |  | rs15795 | unknown |
|  |  | rs3700 | unknown |
|  |  | rs9125 | unknown |
|  |  | rs412790 | unknown |
|  |  | rs1263663 | unknown |
|  |  | rs1263664 | unknown |
|  | TCRB | rs7357130 | unknown |
|  |  | rs965274 | unknown |
|  |  | rs1014957 | unknown |
|  |  | rs17162994 | intron-variant |
|  |  | rs2960760 | intron-variant |
|  |  | rs12674023 | intron-variant,nc-transcript-variant^ǂ^ |
|  |  | rs2156936 | intron-variant |
|  |  | rs1155458 | unknown |
|  |  | rs6958838 | unknown |
|  |  | rs2040369 | unknown |
|  |  | rs7793482 | unknown |
|  |  | rs7778566 | unknown |
|  |  | rs2011310 | unknown |
|  |  | rs361445 | unknown |
|  |  | rs10487531 | unknown |
|  |  | rs1882723 | unknown |
|  |  | rs6979469 | unknown |
|  |  | rs17232 | unknown |
|  |  | rs10276363 | unknown |
|  |  | rs10260565 | unknown |
|  |  | rs2040366 | unknown |
|  |  | rs10216024 | unknown |
|  |  | rs6976636 | unknown |
|  |  | rs11768792 | unknown |
|  |  | rs2213192 | unknown |
|  |  | rs6961143 | unknown |
|  |  | rs975494 | unknown |
|  |  | rs6950275 | unknown |
|  |  | rs17274 | unknown |
|  |  | rs17248 | unknown |
|  |  | rs11768398 | unknown |
|  |  | rs1573618 | unknown |
|  |  | rs17304 | unknown |
|  |  | rs2855896 | unknown |
|  |  | rs2855882 | unknown |
|  |  | rs361489 | unknown |
|  |  | rs949425 | unknown |
|  |  | rs6974518 | unknown |
|  |  | rs7384887 | unknown |
|  |  | rs2285027 | unknown |
|  |  | rs6943492 | unknown |
|  |  | rs2078176 | unknown |
|  |  | rs6975391 | unknown |
|  |  | rs10260647 | upstream-variant-2KB |
|  |  | rs2156966 | unknown |
|  |  | rs17837475 | unknown |
|  |  | rs6946770 | unknown |
|  |  | rs17251 | unknown |
|  |  | rs4726571 | unknown |
|  |  | rs4726572 | unknown |
|  |  | rs6959895 | unknown |
|  |  | rs10273639 | intron-variant,upstream-variant-2KB^ǂ^ |
|  |  | rs2734222 | unknown |
|  |  | rs2734224 | unknown |
|  |  | rs2367486 | unknown |
|  |  | rs3134902 | unknown |
|  |  | rs12539089 | unknown |
|  |  | rs1800907 | unknown |
|  |  | rs11327 | unknown |
|  |  | rs3134906 | unknown |
|  | TCRD | rs11846244 | intron-variant |
|  |  | rs6572449 | intron-variant,upstream-variant-2KB^ǂ^ |
|  |  | rs2141988 | upstream-variant-2KB |
|  |  | rs3811232 | unknown |
|  |  | rs10873018 | unknown |
|  |  | rs12147516 | unknown |
|  |  | rs10483273 | unknown |
|  |  | rs11848747 | unknown |
|  |  | rs11850145 | unknown |
|  |  | rs2242545 | unknown |
|  |  | rs1882704 | unknown |
|  |  | rs1882705 | downstream-variant-500B |
|  |  | rs2733777 | intron-variant,nc-transcript-variant^ǂ^ |
|  |  | rs11622344 | intron-variant,nc-transcript-variant^ǂ^ |
|  | TCRG | rs2240825 | unknown |
|  |  | rs11971259 | downstream-variant-500B |
|  |  | rs28677126 | utr-variant-3-prime |
|  |  | rs2240827 | intron-variant |
|  |  | rs12154478 | intron-variant |
|  |  | rs1860517 | intron-variant |
|  |  | rs10441090 | intron-variant |
|  |  | rs2191311 | intron-variant |
|  |  | rs2248839 | intron-variant |
|  |  | rs2736973 | intron-variant |
|  |  | rs2392546 | intron-variant |
|  |  | rs2736969 | intron-variant |
|  |  | rs6953248 | unknown |
|  |  | rs11769443 | unknown |
|  |  | rs11765884 | unknown |
|  |  | rs718880 | unknown |
|  |  | rs2240850 | unknown |
|  |  | rs17496969 | unknown |
|  |  | rs2191312 | unknown |
|  |  | rs733905 | upstream-variant-2KB |
|  |  | rs2735143 | intron-variant |
|  | TCRGV | rs6953248 | unknown |
|  |  | rs11769443 | unknown |
|  |  | rs11765884 | unknown |
| Akt | AKT1 | rs2494738 | intron-variant |
|  |  | rs2494739 | intron-variant |
|  |  | rs2494743 | intron-variant |
|  | AKT2 | rs2304186 | utr-variant-3-prime |
|  |  | rs4803320 | intron-variant |
|  |  | rs892120 | intron-variant |
|  |  | rs3730256 | intron-variant |
|  |  | rs1991823 | intron-variant |
|  | AKT3 | rs884328 | intron-variant |
|  |  | rs3006927 | intron-variant |
|  |  | rs3006928 | downstream-variant-500B,intron-variant^ǂ^ |
|  |  | rs9428576 | intron-variant,upstream-variant-2KB^ǂ^ |
|  |  | rs1058304 | intron-variant,utr-variant-3-prime^ǂ^ |
|  |  | rs12140414 | intron-variant |
|  |  | rs10927035 | intron-variant |
|  |  | rs1121276 | intron-variant |
|  |  | rs12144559 | intron-variant |
|  |  | rs10927040 | intron-variant,upstream-variant-2KB^ǂ^ |
|  |  | rs3856231 | intron-variant |
|  |  | rs10927046 | intron-variant |
|  |  | rs10754807 | intron-variant |
|  |  | rs12117580 | intron-variant |
|  |  | rs10803152 | intron-variant |
|  |  | rs4658588 | intron-variant |
|  |  | rs2125230 | intron-variant |
|  |  | rs9782883 | intron-variant |
|  |  | rs10803155 | intron-variant |
|  |  | rs4553169 | intron-variant |
|  |  | rs971285 | intron-variant |
|  |  | rs12048930 | intron-variant |
|  |  | rs10733129 | intron-variant |
|  |  | rs6703013 | intron-variant |
|  |  | rs10157763 | intron-variant |
| Fcer1 | FCER1A | rs2427825 | intron-variant |
|  |  | rs7548864 | intron-variant |
|  |  | rs12119226 | intron-variant |
|  |  | rs2252226 | intron-variant |
|  |  | rs7549785 | utr-variant-3-prime |
|  | FCER1G | rs1136224 | upstream-variant-2KB,utr-variant-3-prime^ǂ^ |
|  |  | rs11587213 | upstream-variant-2KB |
|  |  | rs12094497 | intron-variant |
|  |  | rs2070902 | intron-variant |
|  |  | rs4489574 | unknown |
|  | MS4A2 | rs573790 | upstream-variant-2KB |
|  |  | rs1441586 | intron-variant,upstream-variant-2KB,utr-variant-5-prime^ǂ^ |
|  |  | rs2847666 | intron-variant |
|  |  | rs502581 | intron-variant |
|  |  | rs2847655 | utr-variant-3-prime |
| Cg | CGA | rs9359730 | unknown |
|  |  | rs1055409 | utr-variant-3-prime |
|  |  | rs9342104 | intron-variant |
|  | CGB | rs1056917 | downstream-variant-500B,synonymous-codon^ǂ^ |
|  |  | rs7251022 | nc-transcript-variant,upstream-variant-2KB^ǂ^ |
|  |  | rs3810177 | nc-transcript-variant,upstream-variant-2KB^ǂ^ |
|  |  | rs4002419 | upstream-variant-2KB |
|  |  | rs7251022 | nc-transcript-variant,upstream-variant-2KB^ǂ^ |
|  |  | rs11669977 | downstream-variant-500B,utr-variant-5-prime^ǂ^ |
| Interferon alpha | CGB1 | rs1056917 | downstream-variant-500B,synonymous-codon^ǂ^ |
|  |  | rs7251022 | nc-transcript-variant,upstream-variant-2KB^ǂ^ |
|  |  | rs3810177 | nc-transcript-variant,upstream-variant-2KB^ǂ^ |
|  |  | rs4002419 | upstream-variant-2KB |
|  |  | rs7251022 | nc-transcript-variant,upstream-variant-2KB^ǂ^ |
|  |  | rs11669977 | downstream-variant-500B,utr-variant-5-prime^ǂ^ |
|  | CGB2 | rs1056917 | downstream-variant-500B,synonymous-codon^ǂ^ |
|  |  | rs7251022 | nc-transcript-variant,upstream-variant-2KB^ǂ^ |
|  |  | rs3810177 | nc-transcript-variant,upstream-variant-2KB^ǂ^ |
|  |  | rs4002419 | upstream-variant-2KB |
|  |  | rs7251022 | nc-transcript-variant,upstream-variant-2KB^ǂ^ |
|  |  | rs11669977 | downstream-variant-500B,utr-variant-5-prime^ǂ^ |
|  | CGB5 | rs1056917 | downstream-variant-500B,synonymous-codon^ǂ^ |
|  |  | rs7251022 | nc-transcript-variant,upstream-variant-2KB^ǂ^ |
|  |  | rs3810177 | nc-transcript-variant,upstream-variant-2KB^ǂ^ |
|  |  | rs4002419 | upstream-variant-2KB |
|  |  | rs7251022 | nc-transcript-variant,upstream-variant-2KB^ǂ^ |
|  |  | rs11669977 | downstream-variant-500B,utr-variant-5-prime^ǂ^ |
|  | CGB7 | rs1056917 | downstream-variant-500B,synonymous-codon^ǂ^ |
|  |  | rs7251022 | nc-transcript-variant,upstream-variant-2KB^ǂ^ |
|  |  | rs3810177 | nc-transcript-variant,upstream-variant-2KB^ǂ^ |
|  |  | rs4002419 | upstream-variant-2KB |
|  |  | rs7251022 | nc-transcript-variant,upstream-variant-2KB^ǂ^ |
|  |  | rs11669977 | downstream-variant-500B,utr-variant-5-prime^ǂ^ |
|  | CGB8 | rs1056917 | downstream-variant-500B,synonymous-codon^ǂ^ |
|  |  | rs7251022 | nc-transcript-variant,upstream-variant-2KB^ǂ^ |
|  |  | rs3810177 | nc-transcript-variant,upstream-variant-2KB^ǂ^ |
|  |  | rs4002419 | upstream-variant-2KB |
|  |  | rs7251022 | nc-transcript-variant,upstream-variant-2KB^ǂ^ |
|  |  | rs11669977 | downstream-variant-500B,utr-variant-5-prime^ǂ^ |
|  | IFNA1 | rs2939 | utr-variant-3-prime |
|  | IFNA2 | rs615544 | unknown |
|  |  | rs10120977 | utr-variant-3-prime |
|  | IFNA4 | rs2019226 | unknown |
|  |  | rs2007448 | unknown |
|  | IFNA5 | rs7021906 | unknown |
|  | IFNA6 | rs10119678 | unknown |
|  |  | rs614541 | upstream-variant-2KB |
|  | IFNA7 | rs4977686 | unknown |
|  | IFNA8 ^Ɨ^ | - | - |
|  | IFNA10 | rs4977686 | unknown |
|  | IFNA13 | rs2480927 | downstream-variant-500B |
|  | IFNA14 ^Ɨ^ | - | - |
|  | IFNA16 | rs9333362 | unknown |
|  |  | rs3919593 | utr-variant-3-prime |
|  |  | rs7033839 | unknown |
|  | IFNA17 | rs9333362 | unknown |
|  |  | rs3919593 | utr-variant-3-prime |
|  |  | rs7033839 | unknown |
|  | IFNA21 | rs10491569 | intron-variant |

* The IPA-identified molecules can include sub-members, and also, some genes may belong to more than one molecule. Therefore, in the Table, there are duplicated genes and SNPs.

ǂ A given SNP can have more than one function when two or more genes overlap or when there is alternative splicing.

Ɨ No SNP available for the gene on the Illumina HumanOmniExpress-12v1 platform.
